# Neutralization and enhancement of trans infection by erythrocyte-bound HIV with antibodies and complement

**DOI:** 10.1186/1742-4690-9-S2-P49

**Published:** 2012-09-13

**Authors:** Z Beck, GR Matyas, VR Polonis, CR Alving

**Affiliations:** 1 HJF / WRAIR / US Military HIV Research Program, Silver Spring, MD, USA; 2 Walter Reed Army Institute of Research, Silver Spring, MD, USA

## Background

Antibodies to HIV envelope glycoproteins, with or without neutralization when assayed in standard neutralization assays, are reported to have potential to exert either inhibitory or enhancing effects through interactions with complement and/or Fc receptors. Although most of the standard neutralization assays use free virus rather than carrier-bound HIV as the infectious agent; a cell line as the target for infection; and no complement (C), these conditions may not reflect in vivo events that would include antibody-dependent innate effector mechanisms.

## Methods

We investigated the Fc-receptor-mediated and the complement-mediated antibody-dependent enhancement in trans infection neutralization assays using two broadly neutralizing monoclonal antibodies, 4E10 and b12; primary HIV; PBMC target cells naturally expressing Fc receptors and complement receptors; and with or without complement.

## Results

In the absence of complement, the 4E10 mAb did not neutralize erythrocyte-bound HIV, and the b12 mAb neutralized erythrocyte-bound HIV less effective than the free virus. At low concentrations, 4E10 caused enhancement of infection. In contrast, in the presence of complement, 4E10 neutralized erythrocyte-bound HIV, or caused enhancement of trans infection of erythrocyte-bound HIV, depending on the mechanism of binding of HIV to erythrocytes.

## Conclusion

Our results are consistent with the concept that the binding of HIV to erythrocytes represents a mechanism of establishing a “safe harbor” for HIV from the adverse effects of antibodies and complement that might otherwise be detrimental to free circulating HIV. Our data shows that broadly neutralizing antibody 4E10 can cause both C-mediated neutralization and enhancement of trans infection. This suggests that erythrocyte-bound HIV-1 serves as a sort of battleground between neutralization and enhancement by antibodies in the presence of C. Because of this, we propose that induction of neutralizing vs. enhancing antibodies can only be differentiated by utilization of a trans infection neutralization assay that might yield more relevant results for in vivo conditions.

